# Predicted binding site information improves model ranking in protein docking using experimental and computer-generated target structures

**DOI:** 10.1186/s12900-015-0050-4

**Published:** 2015-11-23

**Authors:** Surabhi Maheshwari, Michal Brylinski

**Affiliations:** Department of Biological Sciences, Louisiana State University, Baton Rouge, LA 70803 USA; Center for Computation & Technology, Louisiana State University, Baton Rouge, LA 70803 USA

**Keywords:** Protein-protein interactions, Protein docking, Contact-based symmetry, Protein models, *e*Rank^PPI^, *e*FindSite^PPI^, ZDOCK, ZRANK

## Abstract

**Background:**

Protein-protein interactions (PPIs) mediate the vast majority of biological processes, therefore, significant efforts have been directed to investigate PPIs to fully comprehend cellular functions. Predicting complex structures is critical to reveal molecular mechanisms by which proteins operate. Despite recent advances in the development of new methods to model macromolecular assemblies, most current methodologies are designed to work with experimentally determined protein structures. However, because only computer-generated models are available for a large number of proteins in a given genome, computational tools should tolerate structural inaccuracies in order to perform the genome-wide modeling of PPIs.

**Results:**

To address this problem, we developed *e*Rank^PPI^, an algorithm for the identification of near-native conformations generated by protein docking using experimental structures as well as protein models. The scoring function implemented in *e*Rank^PPI^ employs multiple features including interface probability estimates calculated by *e*FindSite^PPI^ and a novel contact-based symmetry score. In comparative benchmarks using representative datasets of homo- and hetero-complexes, we show that *e*Rank^PPI^ consistently outperforms state-of-the-art algorithms improving the success rate by ~10 %.

**Conclusions:**

*e*Rank^PPI^ was designed to bridge the gap between the volume of sequence data, the evidence of binary interactions, and the atomic details of pharmacologically relevant protein complexes. Tolerating structure imperfections in computer-generated models opens up a possibility to conduct the exhaustive structure-based reconstruction of PPI networks across proteomes. The methods and datasets used in this study are available at www.brylinski.org/erankppi.

## Background

Most proteins work by interacting with other proteins to fulfill their molecular functions, therefore, quaternary assemblies are the key components of the vast majority of biological processes. Consequently, the structural characterization of protein-protein complexes provides valuable insights into protein function and association mechanisms, immensely contributing to the understanding of cellular interaction networks. The knowledge of atomic-level details of protein-protein interactions (PPIs) is required for a number of practical applications, for instance, it is critical for the design of therapeutics targeting protein interfaces [1, 2]. X-ray crystallography and NMR spectroscopy are the most widely used experimental techniques to determine protein complex structures. Nonetheless, these methods cannot keep pace with the rapidly growing number of protein interactions identified by high-throughput approaches such as yeast two-hybrid [3] and affinity purification techniques (co-immunoprecipitation [4], tandem affinity purification [5]) followed by mass spectrometry. The low stability of many complexes as well as significant efforts and high costs associated with experiments certainly impede the systems-level exploration of the molecular structures of protein assemblies. On that account, computational tools for PPI structure modeling bridge the gap between the volume of sequence data, the evidence of binary interactions, and the atomic details of pharmacologically relevant protein complexes.

Quaternary structure modeling to find the best relative orientation of monomers forming a stable complex can be performed using template-based or template-free techniques. Template-based methods use the similarity to known complex structures to model the interaction between a given pair target proteins. This strategy involves superposing target proteins onto the identified templates using either global or interfacial structure alignments [6]. For instance, PRISM models quaternary structures by matching target proteins to a template interface selected from a representative database of the experimental structures of PPI complexes [7, 8]. In contrast, template-free approaches do not use any quaternary information from similar protein complexes; instead, these methods perform docking of the tertiary structures of receptor and ligand proteins. A typical docking calculation comprises two successive steps. First, a rigid-body sampling of six translational and rotational degrees of freedom generates a large set of candidate dimer conformations, in which the constituent monomers are in contact avoiding steric clashes. In the second step, a scoring function is used to rank the disparate collection of docked poses in order to identify near-native models. Current docking algorithms employ a variety of conformational search techniques including a fast Fourier transform [9–11], Monte Carlo methods [12], and the geometric hashing [13, 14]; for recent reviews see [15–17]. Significant efforts have also been devoted to develop reliable scoring functions, many of which assess the stability of the assembled dimers by combining multiple scoring terms such as the geometric shape [18–21], chemical and electrostatic complementarity [22–26]. Nevertheless, despite the advances in pose prediction and scoring, docking programs still face significant difficulties in identifying the best solution from a pool of candidates generated through conformational sampling [22, 27]. Therefore, the development of new approaches to more reliably distinguish between near-native and decoy conformations represents a practical strategy to improve the accuracy of protein docking.

To address the problem of model scoring, the prediction of protein quaternary structures is often supported by a variety of experimental and computational data [28–30]. Several strategies to incorporate experimental data in protein docking have been developed. For instance, upper bounds for distances between residues in interacting protein chains can be identified by NMR spectroscopy [31] and chemical crosslinking [32]. Moreover, simultaneous screening for mutations that disrupt yeast two-hybrid interactions was proposed to identify critical interface residues for multiple interacting partners [33]. Experimental data can be subsequently transformed into distance constrains to narrow the search space and to guide the selection of docking poses [34, 35]. Indeed, data-driven docking has been demonstrated to considerably improve the accuracy of dimer structure modeling [36], nonetheless, a limited availability of experimental data remains the major drawback of large-scale investigations of PPI networks. Although computational methods for interface residue prediction [37, 38] can support the complex assembly through PPI prediction-driven docking strategies, [38, 39] the predicted PPI site information is not always accurate leading to spurious results generated by a misguided conformational sampling.

Interaction symmetry is another commonly used form of constraints to model homo-oligomeric complexes. Symmetry is a prevalent feature of the global arrangement between subunits in homo-oligomer complexes formed by two or more identical protein chains. Homo-dimers are important parts of biochemical pathways that are found to occur more frequently than by chance [40]. Approximately 50-70 % of the available datasets comprise homo-oligomers whose structural symmetry is remarkably well conserved [40–43]. The symmetric organization of proteins is known to confer structural and functional advantages providing stability, control over accessibility and specificity of active sites [44]. It also provides the ability to avoid unwanted aggregation, which is responsible for a number of pathological conditions, such as Alzheimer’s and prion diseases [45, 46]. Furthermore, the symmetric self-association provides an opportunity for cooperative interactions and multivalent binding [47]. Since the cyclic symmetry containing a single rotational axis is the most common type of regularity observed in protein quaternary structures, symmetrical docking a priori restricts the conformational search space only to symmetric transformations [10, 48].

In recent years, a two-stage ranking strategy has gained significant attention. Here, a standard protocol is first employed to rapidly scan for putative dimer conformations and to identify a subset of plausible candidates. Subsequently, an additional scoring system is used to re-rank the docked conformations in order to improve the ranking of near-native poses. These methods integrate a variety of features including sophisticated energy calculations, experimental and predicted binding site locations, statistical potentials derived from databases of complex structures, and evolutionary information [28, 49]. For instance, ZRANK [50] combines van der Waals, electrostatic and desolvation energy terms to re-rank the initial docking predictions generated by ZDOCK [9], whereas DECK [51] employs a distance and environment dependent knowledge-based potential to refine predictions from GRAMMX [52]. Furthermore, the accuracy of HADDOCK [29] was improved by applying a scoring function based on a Voronoi tessellation of protein structures and machine learning [53]. Other examples include T-PioDock [54], which uses interface prediction to assist the ranking of docked poses, and ClusPro [55] that re-ranks the top 2000 solutions generated either by ZDOCK or DOT [56] using a greedy clustering technique. Most of the available re-ranking protocols were designed and subsequently benchmarked using the experimentally determined structures in their bound and unbound conformational state. Since the structure-based reconstruction of across-proteome interaction networks involves docking of various quality homology models, re-ranking strategies should ideally tolerate inaccuracies in the atomic coordinates of interacting monomers.

In that regard, we developed *e*Rank^PPI^, an algorithm for the selection of correct docking conformations constructed by protein docking using not only experimental monomer structures but also protein models. A scoring function implemented in *e*Rank^PPI^ combines in a novel way certain features such as residue-level interface probabilities estimated by *e*FindSite^PPI^ [57], protein docking potentials [58], and a new contact-based symmetry score. Although, the predicted interface location was already successfully employed to improve the ranking accuracy for docked conformations [54], most previously reported benchmarking calculations were carried out against relatively small datasets of experimental structures [59–61]. In contrast, in this study, we perform a comprehensive analysis using non-redundant and representative sets of crystal structures as well as various quality protein models. In large-scale benchmarks using homo- and hetero-complexes, the accuracy of *e*FindSite^PPI^ is compared to state-of-the-art scoring methods.

## Methods

### Datasets and tools

The algorithm for the re-ranking of docking models is trained and tested on the BM1905 dataset of 1905 proteins, which was compiled previously to evaluate the accuracy of interface residue prediction [57]. This dataset contains experiment target structures (BM1905C) as well as high- and moderate- quality models (BM1905H and BM1905M, respectively). The quality of computer-generated models was assessed by TM-score [62], which ranges from 0 to 1 with values ≥0.4 indicating a significant structure similarity to the native protein. BM1905M and BM1905H datasets comprise models whose TM-score is in the range of 0.4–0.7 and 0.7–0.9 respectively. Furthermore, the BM1905 dataset contains 1755 homo-dimers (BM1755) and 150 hetero-dimers (BM150).

ZDOCK [9] version 3.0.2 is used to generate rigid-body docking conformations with the default search parameters. It has consistently been among the best performing algorithms in the Critical Assessment of Prediction of Interactions (CAPRI) [27, 63–66], a community-wide project assessing the accuracy of protein-protein docking algorithms. ZDOCK employs a fast Fourier transform (FFT) correlation-based method, which performs a systematic search in the six-dimensional space created by 3 rotational and 3 translational degrees of freedom. Docking conformations are predicted based on the desolvation and electrostatics contributions to the complex formation as well as the pairwise shape complementarity. Prior to docking, both the receptor and ligand structures are randomly translated and rotated to avoid any bias towards initial orientations. We collect 2000 highest scoring conformations reported by ZDOCK for each protein.

In this study, putative interfacial sites are predicted for the benchmarking receptors by *e*FindSite^PPI^ [57], a recently developed structure/evolution-based approach to detect interface residues. *e*FindSite^PPI^ exploits a general tendency of the location and geometry of binding sites to be highly conserved in evolutionarily weakly related dimer proteins. It employs a collection of effective algorithms, including meta-threading by *e*Thread [67], structure alignments by Fr-TM-align [62], and machine learning using Support Vector Machines (SVMs) and a Naïve Bayes Classifier (NBC) [68]. Each residue in the query protein is assigned a probability to be at the interface using residue-level attributes in combination with sequence and structure conservation scores derived from evolutionarily related templates.

### Training attributes

*e*Rank^PPI^ developed in this study employs a series of attributes to re-rank docking conformations, including residue-level interface probabilities, protein docking contact potentials, and energy-based scores. The training and evaluation is performed separately for homo- and hetero-dimers as the modeling of homo-complex structures additionally takes account of symmetry constraints. Individual features are described below.

#### Interface scores

*e*Rank^PPI^ incorporates interface probability estimates for the receptor protein. We use probability scores assigned to each residue in the target protein by *e*FindSite^PPI^ to estimate the likelihood to be at the protein-protein interface. Interfacial residues in docking models constructed by ZDOCK are identified by iAlign [69], which uses a distance-based criterion to identify the interface in a given multimer structure. The interface score is the sum of probabilities calculated over interface residues; two scores are computed using SVC and NBC. In general, these scores favor docking conformations with a substantial coverage of surface regions assigned a high interfacial probability by *e*FindSite^PPI^.

#### Protein docking potential

In addition to the interface scores, we employ a protein docking potential previously developed using a linear programming technique [58]. In this model, the side chain center of mass, the backbone carbonyl oxygen, and the amide group are considered interaction sites for each residue. Inter-residue contacts are defined using distance thresholds of 6.8 Å, 4.0 Å and 5.6 Å for side chain, backbone and backbone/side chain sites, respectively. Two hundred fifty-three independent pairwise parameters were optimized in order to efficiently discriminate between hits and non-hits across protein-protein ensembles constructed by rigid-body docking.

#### ZDOCK energy score

Conformational ensembles of putative dimers are constructed by ZDOCK, as described above. The scoring function implemented in ZDOCK is a linear weighted sum of van der Waals attractive and repulsive energies, short- and long-range attractive and repulsive electrostatic energies, and desolvation. The optimal set of weight factors that maximizes the discriminatory capabilities of ZDOCK was obtained by training the scoring function on the Benchmark 1.0 set [70], followed by a cross-validation against non-homologous cases selected from the Benchmark 2.0 set [71]. We use the total energy score reported by ZDOCK as one of the components of the scoring function in *e*Rank^PPI^.

#### Symmetry score

**Fig. 1:**
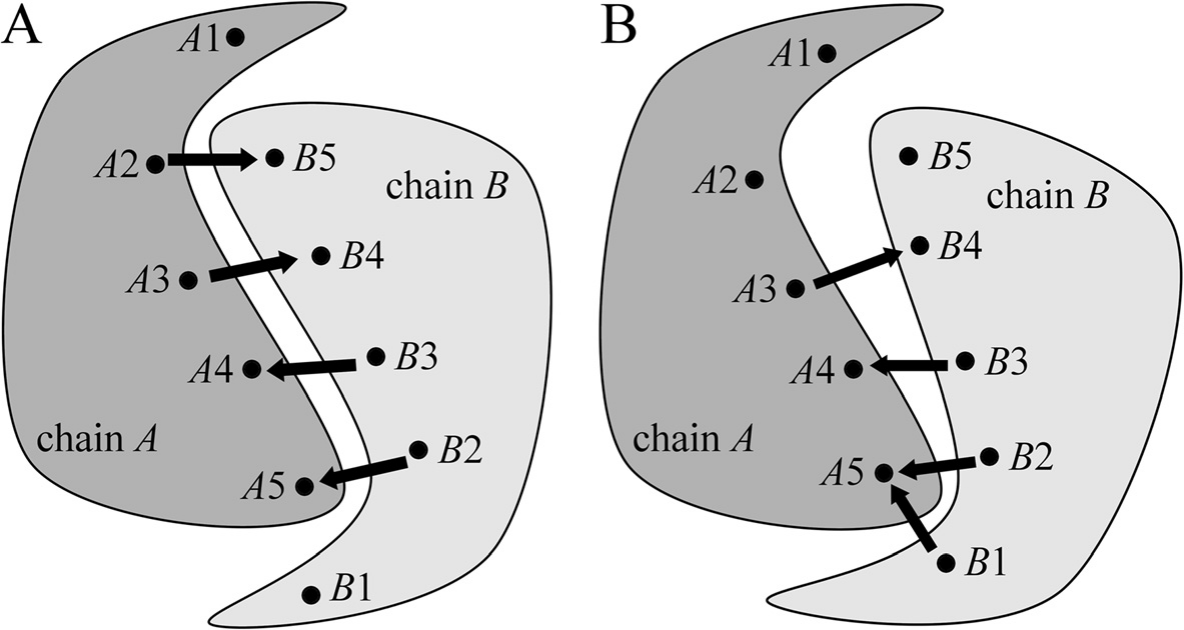
Calculation of the contact-based symmetry score. The schematics illustrate pairwise residue contacts in **a** a completely symmetric dimer and **b** a partially symmetric dimer. *Ax* → *By* denotes that the residue number *x* in chain *A* is in contact with the residue number *y* in chain *B*

The vast majority of homo-dimers form symmetric interfaces, therefore, we include the deviation from an ideal point group cyclic symmetry in the scoring function to re-rank the homo-complex models. Specifically, we developed a new metric to measure the degree of symmetry at the protein-protein interface, called the contact-based symmetry score (CBS). Figure 1 shows two complexes of identical protein chains *A* (dark gray) and *B* (light gray) with residues numbered as *A*1, *A*2 … *A*5 and *B*1, *B*2 … *B*5, respectively. A complex shown in Fig. 1a is perfectly symmetrical at the interface, whereas that presented in Fig. 1b deviates from the ideal symmetry. To quantify this deviation, we first find all inter-residue contacts, defined as those residue pairs, for which any two non-hydrogen atoms are within a distance of 10 Å. For example, in the complex shown in Fig. 1b, interacting residue pairs are *A*3 : *B*4, *A*4 : *B*3, *A*5 : *B*2, and *A*5 : *B*1; the notation *Ax* : *By* means that the residue number *x* in chain *A* is in contact with the residue number *y* in chain *B* where *x* ≠ *y*. Next, we divide residue pairs into two sets, *S*1 and *S*2, so that *S*1 contains pairs with *x* < *y* and *S*2 contains pairs with *x* > *y*. For the complex shown in Fig. 1b, this gives us *S*1 = {*A*3 : *B*4} and *S*2 = {*A*4 : *B*3, *A*5 : *B*2, *A*5 : *B*1}. Finally, the CBS score is calculated as the Jaccard index to measure the similarity between *S*1 and *S*2:

1 $$ CBS=\frac{\left|S1\cap S2\right|}{\left|S1\cup S2\right|} $$

Essentially, the Jaccard index is a ratio of the intersection and the union between the two sets of interacting residue pairs, where *Ax* : *By* is considered a match for *Ay* : *Bx*. CBS ranges from 1 for perfectly symmetrical interfaces to 0 for completely asymmetrical complexes. For example, CBS scores calculated for homo-dimers shown in Fig. 1a and b, are 1 (perfect symmetry) and 1/3 (one-third of a perfect symmetry), respectively. The CSB scores are used only for homo-dimers, therefore five features are computed by *e*Rank^PPI^ for homo-complexes, whereas four features are used for hetero-dimers.

### Supervised learning

The scoring function implemented in *e*Rank^PPI^ is trained and cross-validated on docking ensembles generated by ZDOCK separately for the BM1755C and BM150C datasets. Specifically, we calculate the set of either five (homo-dimers) or four (hetero-dimers) attributes for statistical learning in order to rank individual conformations so that near-native structures are assigned lower ranks compared to decoy complexes. The learning procedure is supervised by an iRMSD-based ranking, where the iRMSD is a root-mean-square deviation from the experimental complex structure calculated over the Cα atoms of interface residues. Consequently, the ranking problem can be formulated as the prediction of iRMSD values from individual attributes using a regression analysis. We note that all benchmarking calculations are carried out using a two-fold cross validation protocol by randomly splitting dataset proteins to avoid memorization effects in machine learning. We tested several linear and non-linear models and found that for homo-dimers, Support Vector Regression, epsilon-SVR, with a radial basis function kernel from the LIBSVM version 3.14 [72] yields the best performance. Because of a much smaller dataset size, we use a linear regression (LR) model [73] for hetero-dimers. Furthermore, individual attributes are standardized independently for each target complex in order to account for proteins of different lengths forming interfaces of different sizes. Specifically, a raw attribute value *x* is converted to the standard score (*Z*-score) as follows:

2 $$ Z\hbox{-} \mathrm{score}=\frac{x-\overline{x}}{\sigma_x} $$

where $\overline{x}$ is the mean attribute value calculated across the dimer ensembles generated for a given pair of target proteins by ZDOCK, and *σ*_*x*_ is the corresponding standard deviation.

### Evaluation of docking predictions

The quality of model dimer structures is assessed using two metrics, iRMSD and a contact-based score. The iRMSD is a standard evaluation measure in CAPRI corresponding to the interface Cα-RMSD between a ligand in the predicted complex and the ligand in the experimental structure upon the superposition of the receptor structures. In iRMSD calculations, interface residues are defined as those having at least one atom within 10 Å from any atom in the other protein chain. In addition to the iRMSD, the accuracy of complex structures can be evaluated at the level of pairwise residue contacts. Previously, *f*_nat_ and *f*_non-nat_ have been used to assess the quality of predicted interface interactions [74]. The former is defined as the number of correct (native) residue-residue contacts in the predicted complex divided by the total number of contacts in the experimental structure, whereas the latter is the fraction of non-native contacts in the predicted complex divided by the total number of contacts in that model. Note that *f*_nat_ alone may be insufficient to reliably assess the model accuracy because of possible over-predicted interface contacts, which are revealed by *f*_non-nat_. Because, a single metric is more convenient to evaluate the accuracy of protein docking predictions, we formulated a Pairwise Contact Score (PCS). Similar to the iRMSD, pairs of residues on different chains are in contact if any of their atoms are within 10 Å from each other. PCS employs Matthew’s correlation coefficient (MCC) to evaluate the strength of a correlation between the predicted and actual classes:

3 $$ MCC=\frac{TP\times TN-FP\times FN}{\sqrt{\left(TP+FP\right)\left(TP+TN\right)\left(FP+FN\right)\left(TN+FN\right)}} $$

where *TP* (True Positives), *FN* (False Negatives) and *FP* (False Positives) is the number of correctly predicted, under-, and over-predicted pairwise contacts, respectively. *TN* (True Negatives) is the number of correctly predicted non-contacting residue pairs. Importantly, PCS considers both the accuracy and error rates, and it is less affected by the imbalanced numbers of positives (pairwise interface contacts) and negatives (non-contacting pairs). Theoretically, MCC ranges from −1 to 1, where 1 corresponds to a perfect prediction and −1 is a perfectly inverse prediction; in practice, PCS scores vary from about 0 to 1.

### Assessment of model ranking

Protein docking algorithms typically construct multiple dimer models for a given pair of protein structures. Therefore, a reliable scoring function is critical to rank the predicted models so that near-native structures can be selected from a large set of decoys. In that regard, we evaluate the ranking capability using the following measures:

#### Percentage of successful cases

This metric reports the percentage of docking cases for which at least one hit is ranked within the top 10 models. Hits are defined as those conformations having iRMSD below a given cutoff varying from 0 to 15 Å. In addition to the iRMSD, we also calculate the percentage of successful cases using PCS as the hit criterion where the respective cutoff changes from 1 to 0.

#### Hit count

Hit count gives the average number of hits within the top 10 docking models across the benchmarking dataset. Hits are predictions whose iRMSD is below a given cutoff ranging from 0 to 15 Å. Thus the hit count measures the overall enrichment of the top ranked models with near-native conformations.

#### Success rate

The docking success rate is defined as the percentage of targets for which at least one correct model is ranked within the top *n* conformations, where *n* changes from 1 to 1000. The acceptance criteria for correct predictions are an iRMSD of ≤2.5 Å, ≤8.5 Å and ≤9.5 Å for experimental structures, high- and moderate-quality models, respectively.

## Results

### Symmetry in homo-dimers

**Fig. 2:**
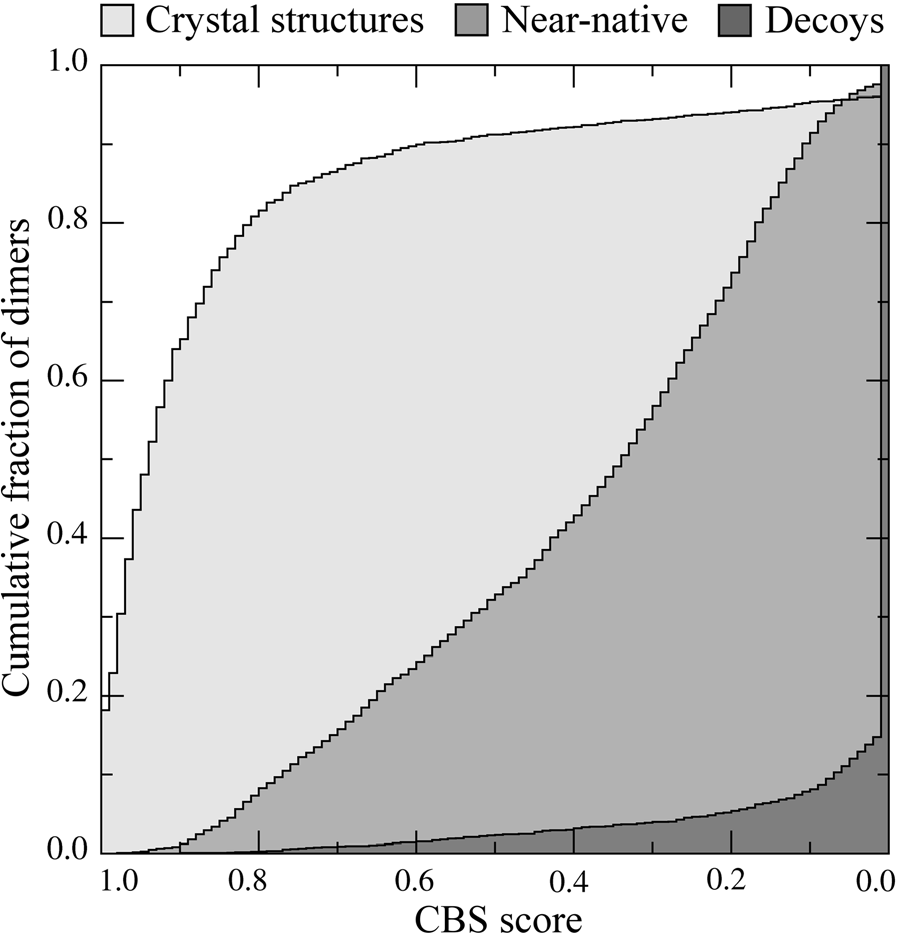
Distribution of contact-based symmetry scores across the BM1755 dataset. The results are presented as cumulative fraction of homo-dimers with a contact-based symmetry (CBS) score larger than or equal to the value displayed on the *x*-axis. CBS quantifies the deviation of a homo-dimer from an ideal cyclic symmetry. Near-native structures and random decoys are those dimer models whose iRMSD from the corresponding experimental complexes is ≤5 Å and >20 Å, respectively

*e*Rank^PPI^ employs a new measure, called CBS, which quantifies the deviation from an ideal cyclic symmetry using inter-residue contacts rather than purely geometrical features. First, we calculated the distribution of CBS scores across the experimental homo-dimer structures from the BM1755C dataset. Figure 2 demonstrates that the fraction of proteins self-interacting through symmetrical interfaces is notably higher than those having an asymmetric arrangement of their quaternary structures. For instance, 86.6 % of the protein complexes have a CBS of ≥0.7, compared to only 8.7 % with a CBS below 0.5. These results concur with previous studies presenting the symmetry as a rule in the global arrangement of homo-dimers [41, 47]. Next, we calculated CBS scores for dimers assembled by ZDOCK. Here, we separately analyze two subsets of models, 2000 randomly selected near-native structures whose iRMSD from the corresponding experimental complexes is ≤5 Å, and 2000 random decoys with an iRMSD of >20 Å. As shown in Fig. 2, the near-native models tend to deviate from an ideal symmetry to a lesser degree compared to decoys; for example, 50 % of near-native structures have a CBS of at least 0.33, whereas only 3.6 % of decoys are found at this CBS threshold.

These findings encouraged us to use the CBS as one of the features to improve the ranking of homo-dimers. As a matter of fact, the concept of symmetry is widely used to construct homo-dimer complexes. Several protein docking programs were developed to model homo-oligomer structures by performing a systematic space search exclusively for symmetric conformations, e.g., M-ZDOCK [10], SymmRef [75] and SymmDock [48, 76]. These programs commonly use the symmetry to narrow the search space, however, *e*Rank^PPI^ employs a different approach. First, it incorporates the deviation from an ideal symmetry as a feature to improve the ranking of near-native models within docking ensembles generated through an unrestricted conformational search. Second, *e*Rank^PPI^ exploits a contacts-based symmetry rather than geometric regularities, which is more suitable for complex assembly using computer-generated monomers whose tertiary structures are somewhat distorted compared to experimental structures. To our knowledge, the pairwise contact-based symmetry is a novel feature used by *e*Rank^PPI^ in the modeling of homo-dimers.

### Quality of predicted binding interfaces

The knowledge of PPI sites can be used to improve the success rate in protein docking [28, 36, 77]. Several groups integrated experimentally determined PPI information into their docking algorithms either to restrict the docking space during pose prediction or to filter the constructed conformations as a post-processing step. Moreover, due to the limited availability of experimental data, predicted PPI sites can be used instead. Nonetheless, the predicted PPI information is not always highly accurate and using erroneous data may lead to failed predictions. Ideally, docking strategies utilizing predicted PPI sites should tolerate to some extent only partially accurate constraints. In *e*Rank^PPI^, we use interface residue prediction by *e*FindSite^PPI^ that produces a continuous range of probability estimates over surface residues in target proteins rather than just a binary classification of interacting and non-interacting residues. These probability estimates are used to calculate the cumulative interface score for a given docking model, which is advantageous over the binary classification as it better tolerates a weaker signal from PPI prediction with moderate and low accuracy.

**Fig. 3:**
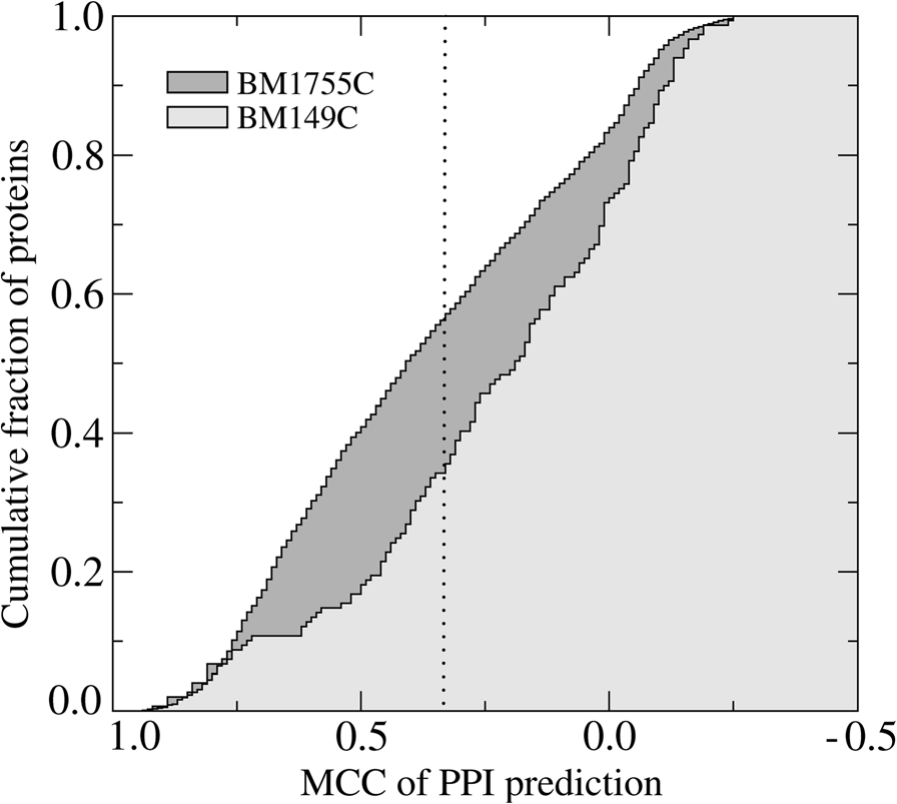
Accuracy of PPI site prediction for the BM1905 dataset. The results are presented as the cumulative fraction of proteins with Matthew’s correlation coefficient (MCC) between predicted and experimental interface residues larger than or equal to the value displayed on the *x*-axis. A dotted vertical line marks an MCC of 0.3

Since the quality of predicted binding interfaces is important for the subsequent modeling of dimer structures, we first inspect the distribution of the PPI prediction accuracy across benchmarking datasets. For each protein target, we calculate Matthew’s correlation coefficient between interface residues in the experimental complex and those predicted by *e*FindSite^PPI^. The results for BM1755C (homo-dimers) and BM149C (hetero-dimers) are presented in Fig. 3. For example, PPI interfaces are predicted with an MCC of ≥0.3 for 58 % and 39 % of BM1755C and BM149C targets, respectively. We note that PPI residues are identified using evolutionarily weakly homologous templates at the 40 % sequence identity threshold. Similar to other template-based PPI residue predictors [78, 79], the overall performance of *e*FindSite^PPI^ for homo-complexes is notably better than that for hetero-complexes, which are underrepresented in the PDB.

**Fig. 4:**
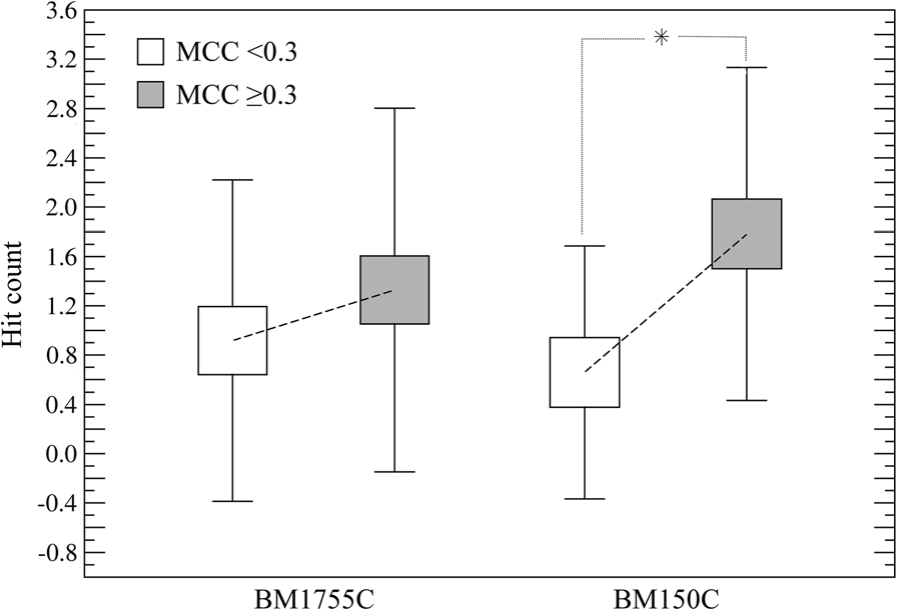
Effect of the PPI prediction accuracy on dimer ranking by *e*Rank^PPI^. The BM1755C and BM58C datasets are divided into two subsets with respect to the accuracy of interface residue prediction (MCC ≥0.3 and MCC <0.3). The average hit count ± standard deviation is then calculated separately for each subset. An asterisk indicates that the ranking capability of *e*Rank^PPI^ for hetero-dimers is significantly affected by the accuracy of PPI residue prediction with a *p*-value of <0.05

Next, we investigate the effect of the PPI prediction accuracy on the quality of dimer models selected by *e*Rank^PPI^ from docking ensembles constructed by ZDOCK. Specifically, we divide each dataset based on the MCC of PPI site prediction using a cutoff of 0.3 and compare the ranking capability of *e*Rank^PPI^. Figure 4 shows the average hit count and the standard deviation calculated at an iRMSD of 2.5 Å for homo-dimers (BM1755C) and hetero-dimers (BM150C). The average hit count for the BM1755C dataset is 1.35 and 0.94 considering those target proteins whose PPI residues are predicted with an MCC of ≥0.3 and <0.3, respectively. For the BM150C dataset, the average hit count is 1.79 at an MCC of ≥0.3 and 0.67 at an MCC of <0.3. To assess the statistical significance of these differences, we calculated the corresponding *p*-values using the Wilcoxon signed-rank test, a non-parametric alternative to the paired Student’s *t*-test [13]. At the 5 % significance level, the accuracy of PPI residue prediction for hetero-dimers affects the ranking capability of *e*Rank^PPI^ with a *p*-value of 0.027. In contrast, a *p*-value of 0.121 indicates that the selection of near-native models for homo-dimers is less affected by the quality of the PPI interfaces predicted by *e*FindSite^PPI^. The main reason for the higher tolerance of inaccurately annotated interface residues for homo-dimers is the additional score, CBS, which helps eliminate the majority of asymmetric decoys.

### Ranking using experimental structures

In order to evaluate the performance of *e*Rank^PPI^, we first re-ranked the top 2000 models assembled by ZDOCK from monomers in their bound conformational state. We use iRMSD and PCS to assess the native-likeness of modeled dimer structures and analyze the results in terms of the percentage of successful cases, the hit count and the success rate. First, we evaluate the ranking capability of *e*Rank^PPI^ compared to ZDOCK and ZRANK against homo-dimers from the BM1755C dataset. Table 1 shows that using *e*Rank^PPI^, at least one model with an iRMSD below 2.5 Å is found within the top 10 ranked conformations for 58.1 % of the benchmarking cases. This performance represents an improvement over ZDOCK and ZRANK, which give the percentage of successful cases of 51.1 and 55.2 % respectively. We also assessed the contribution of the symmetry score to the overall success; removing the symmetry score from the scoring function yields the percentage of successful cases of 56.1 %. Moreover, using PCS with a cutoff of 0.65 as the success criterion, *e*Rank^PPI^ improves model ranking by 17.2 % (8.6 %) with respect to ZDOCK (ZRANK).

**Table 1:** Comparison of the success rates for different scoring functions against experimental target structures

Dataset	Scoring function	Success rate [%]	
BM1755C		iRMSD = 2.5 Å	PCS = 0.65
	*e*Rank^PPI^	58.08	58.86
	ZDOCK	51.13	51.68
	ZRANK	55.18	55.49
BM58C		iRMSD = 2.5 Å	PCS = 0.65
	*e*Rank^PPI^	84.42	84.48
	ZDOCK	67.75	67.24
	ZRANK	75.86	75.86

**Fig. 5:**
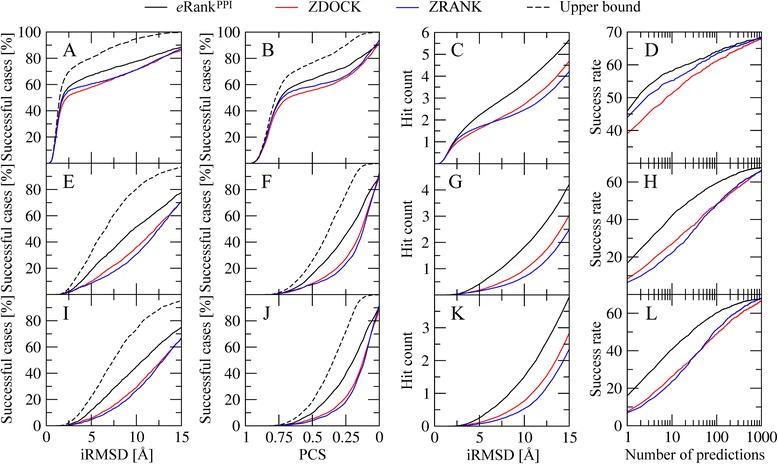
Performance of *e*Rank^PPI^, ZDOCK and ZRANK on the BM1755 dataset. Ranking accuracy is assessed by the percentage of successful cases based on **a**, **e**, **i** iRMSD and **b**, **f**, **j** PCS, **c**, **g**, **k** the hit count, and **d**, **h**, **l** the success rate. Each algorithm is evaluated against **a**-**d** experimental structures, as well as **e**-**h** high-quality and **i**-**l** moderate-quality protein models. Black dashed lines shown for the percentage of successful cases correspond to the upper bound estimated by taking the best of all 2000 models constructed for each target

Further comparison of the overall performance of *e*Rank^PPI^, ZDOCK and ZRANK is shown in Fig. 5. Figure 5a and b demonstrate that the percentage of successful cases within the top 10 conformations for *e*Rank^PPI^ is higher than that for ZDOCK and ZRANK over a range of iRMSD and PCS threshold values used to define correct predictions. The same holds true for the hit count and the success rate; for instance, Fig. 5c shows that using *e*Rank^PPI^ yields an average number of 2.21 hits per target within the top 10 ranked predictions at an iRMSD cutoff of 5 Å, whereas the hit count for ZDOCK and ZRANK is 1.60 and 1.68, respectively. Model ranking by *e*Rank^PPI^ is consistently better than that by ZDOCK and ZRANK not only for the top 10 but also considering lower ranks, which can be evaluated using the success rate shown in Fig. 5d. These results suggest that compared to other algorithms, the scoring function implemented in *e*Rank^PPI^ more reliably identifies near-native models of homo-dimer complexes across docking ensembles.

**Fig. 6:**
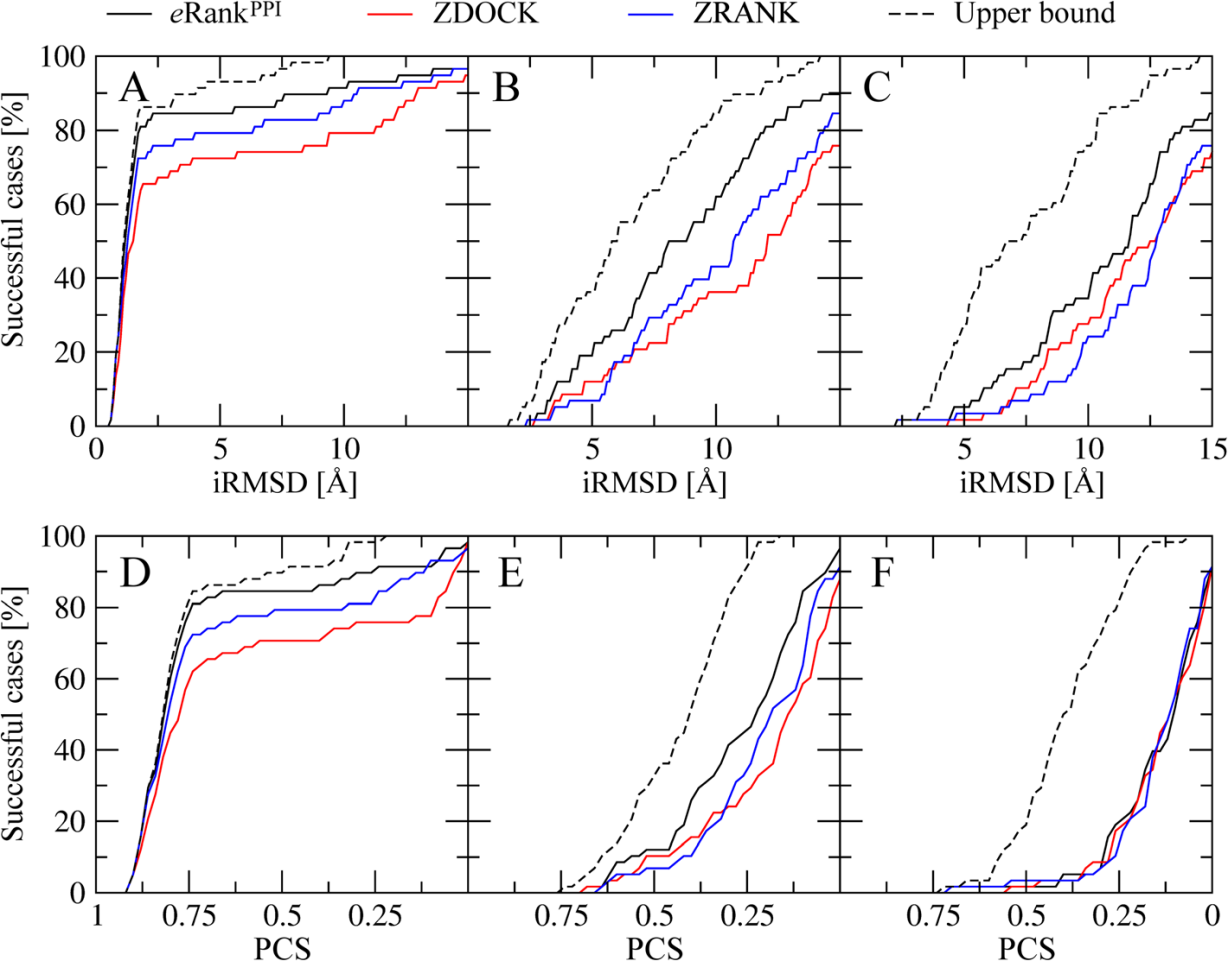
Performance of *e*Rank^PPI^, ZDOCK and ZRANK on the BM58 dataset. Ranking accuracy is assessed by the percentage of successful cases based on **a**-**c** iRMSD and **d**-**f** PCS. Each algorithm is evaluated against **a**, **d** experimental structures, as well as **b**, **e** high-quality and **c**, **f** moderate-quality protein models. Black dashed lines correspond to the upper bound estimated by taking the best of all 2000 models constructed for each target

Next, we turn over to hetero-dimers and compare the performance of *e*Rank^PPI^, ZDOCK and ZRANK for the BM155 dataset. The success rate of ZDOCK, ZRANK and *e*Rank^PPI^ against BM155C targets is 53.7, 67.1 and 58.4, respectively. The analysis of the quality of predicted binding interfaces on the docking accuracy presented above indicates that *e*Rank^PPI^ is sensitive to inaccuracies in PPI annotation for hetero-complexes. Therefore, we use a subset of 58 targets selected from BM155 whose interface residues are predicted with an MCC of ≥0.3; we refer to this dataset as BM58. Figure 6a shows that the ranking capability of *e*Rank^PPI^ for the BM58C dataset is better than that of ZDOCK and ZRANK. For example, Table 1 shows that at an iRMSD threshold of 2.5 Å, the percentage of successful cases for *e*Rank^PPI^, ZDOCK and ZRANK is 84.4, 67.8 and 75.9 % respectively. Similar improvements are observed for the PCS used as the success criterion in Fig. 6d; using *e*Rank^PPI^ improves the ranking by ZDOCK (ZRANK) by 13.8 % (5.2 %). We note that in contrast to homo-dimers, *e*Rank^PPI^ does not improve model ranking for those targets whose binding interfaces are poorly annotated, therefore, a sufficiently high accuracy of PPI residue prediction is critical for the construction of hetero-dimer structures.

### Ranking using computer-generated models

Genome-wide determination of protein interaction networks is an important step in the elucidation of cellular regulatory mechanisms [80, 81]. Although constituent interactions can be modeled through a structure-based dimer assembly, the performance of scoring functions for model selection certainly depends on the quality on input structures. So far, we discussed the ranking of dimer models constructed from experimental monomer structures. Nonetheless, despite the exponential growth of the PDB, experimentally determined structures of a vast majority of gene products are not yet available. This necessitates using computer-generated models in protein docking, however, assuming that a docking program is capable to reliably construct complexes using theoretical monomer structures. Previously, a low-resolution docking method was applied to protein models [82] as a starting point for the subsequent high-resolution refinement to address the challenges of PPI modeling at a proteome-wide scale.

Here, we investigate how different docking scoring strategies cope with inaccuracies in the computer-generated models of query proteins. Undoubtedly, docking using protein models represents a difficult task and the quality of the resulting dimers cannot be higher than the quality of monomer structures. An iRMSD cutoff of 2.5 Å is widely accepted as a criterion for near-native models using experimental structures. However, different threshold values need to be used to evaluate dimer structures assembled from computer-generated models in order to account for distortions in individual monomers. Therefore, we first calculated the distribution of hits with an iRMSD of 2.5 Å across the top 2000 docking models constructed by ZDOCK using experimental monomer structures. A black dashed line in Fig. 5a shows that at least one assembled dimer has an iRMSD of 2.5 Å for about 70 % of the target proteins. We found that an iRMSD cutoff of 8.5 Å (9.5 Å) gives a similar coverage when high- (moderate-) quality models are used in protein docking. Furthermore, we established PCS cutoffs in a similar fashion so that ~70 % of the cases have at least one hit within docking ensembles; the corresponding threshold values are 0.65, 0.30 and 0.25 for crystal structures, high- and moderate-quality models, respectively.

Using these iRMSD and PCS cutoffs to define accurate predictions, we evaluate the ranking capability of *e*Rank^PPI^, ZDOCK and ZRANK on the BM1755H and BM1755M datasets of homo-dimers. Table 2 shows that *e*Rank^PPI^ places at least one model with an iRMSD of ≤8.5 Å (≤9.5 Å) within the top 10 conformations for 42.7 % (42.3 %) of the high- (moderate-) quality models. This performance represents a significant improvement over both ZDOCK and ZRANK, which give the percentage of successful cases of 27.6 % (26.9 %) and 22.5 % (24.6 %), respectively. Furthermore, the overall performance of *e*Rank^PPI^, ZDOCK and ZRANK for homo-dimer targets is compared in Fig. 5. Figure 5e, f, i and j demonstrate that the percentage of successful cases within the top 10 conformations for *e*Rank^PPI^ is closer to the estimated upper limit than for ZDOCK and ZRANK over a range of iRMSD and PCS threshold values defining correct predictions. We note that the black dashed lines in Figs. 5 and 6 represent upper bounds for the docking accuracy calculated by selecting the best dimer from the entire ensemble of 2000 structures constructed by ZDOCK for a given target protein.

**Table 2:** Comparison of the success rates for different scoring functions against high- and moderate-quality protein models

Dataset	Scoring function	Success rate [%]	
BM1755H		iRMSD = 8.5 Å	PCS = 0.30
	*e*Rank^PPI^	42.71	38.23
	ZDOCK	27.61	22.05
	ZRANK	22.55	18.17
BM1755M		iRMSD = 9.5 Å	PCS = 0.25
	*e*Rank^PPI^	42.31	20.68
	ZDOCK	26.99	18.16
	ZRANK	24.60	17.24

Similar performance improvements are observed for the hit count and the success rate. For instance, Fig. 5g and k show that using *e*Rank^PPI^ yields an average number of 1.36 and 1.35 hits per target for the BM1755H and BM1755M datasets at the iRMSD cutoffs of 8.5 and 9.5 Å, respectively. For comparison, the corresponding hit counts for ZDOCK (ZRANK) are only 0.66 (0.69) and 0.46 (0.47). Furthermore, in Fig. 6, we examine the performance of *e*Rank^PPI^, ZDOCK and ZRANK on the BM58H and BM58M datasets of hetero-dimers. For instance, Fig. 6b and c show that the percentage of successful cases at an iRMSD of 8.5 Å (9.5 Å) obtained by *e*Rank^PPI^, ZDOCK and ZRANK for BM58H (BM58M) is 50.0 % (34.4 %), 29.31 % (27.5 %) and 34.5 % (17.2 %) respectively. This comprehensive analysis using various evaluation measures demonstrates that dimer ranking by *e*Rank^PPI^ is consistently better than that by ZDOCK and ZRANK not only using experimental monomer structures, but also computer-generated models.

## Discussion

The identification of near-native conformations across docking ensembles remains a challenging problem in the structure-based modeling of protein-protein interactions. Docking strategies need accurate scoring functions to rank the predicted conformations. Many current approaches employ the geometric, chemical and electrostatic complementarity as well as knowledge-based interaction potentials as components of their scoring functions. In this communication, we describe *e*Rank^PPI^, a new scoring method for protein-protein docking that integrates predicted binding site information, protein docking potentials, energy-based scoring and a contact-based symmetry constraints (for homo-dimers). Although these attributes have been used previously in protein docking, we combined them in *e*Rank^PPI^ as a single, machine learning-based scoring function. The results demonstrate that *e*Rank^PPI^ reliably selects near-native conformations from a large number of decoys generated by ZDOCK [9]. Moreover, comparative benchmarks show that *e*Rank^PPI^ consistently outperforms the state-of-the-art algorithms, ZDOCK and ZRANK, for both homo- and hetero-complexes yielding notably higher hit counts and success rates.

In addition to experimental target structures, we performed a series of benchmarking simulations using computer-generated models. Interestingly, ZRANK performs better than ZDOCK only against experimental target structures. The main reason for this high sensitivity to distortions in target structures is likely a strong dependence on atomic potentials, therefore, ZRANK requires high-quality structural data in order to provide accurate ranking. In contrast, *e*Rank^PPI^ outperforms both ZDOCK and ZRANK not only using experimental structures, but also computer-generated models. This is an important feature of *e*Rank^PPI^ owing to the fact that protein models represent the most challenging targets for molecular docking.

The analysis of the linear regression model used by *e*Rank^PPI^ to rank hetero-dimers shows that the optimized weights for the SVC and NBC interface scores assigned by *e*FindSite^PPI^, the protein-docking potential and the ZDOCK score are 171.9, 891.8, 122.7 and 2.2, respectively. Therefore, the predicted binding site information is a major contributor to the improvement of model ranking in protein docking. Since the success of *e*Rank^PPI^ depends on the accuracy of protein interface prediction, using a robust PPI prediction program is essential. Here, we use *e*FindSite^PPI^, a recently developed template-based approach that effectively exploits the tendency of the location of binding sites to be highly conserved across evolutionarily related protein dimers [57]. *e*FindSite^PPI^ uses the three-dimensional structure of a query protein, evolutionarily remotely related templates and machine learning to predict interfacial sites. It was also shown to outperform several PPI site prediction programs [83]. Also, different from other prediction techniques, *e*FindSite^PPI^ tolerates structural imperfections in computer-generated models. These characteristics make *e*FindSite^PPI^ a preferred PPI predictor to support dimer ranking in across-proteome docking studies using *e*Rank^PPI^.

**Fig. 7:**
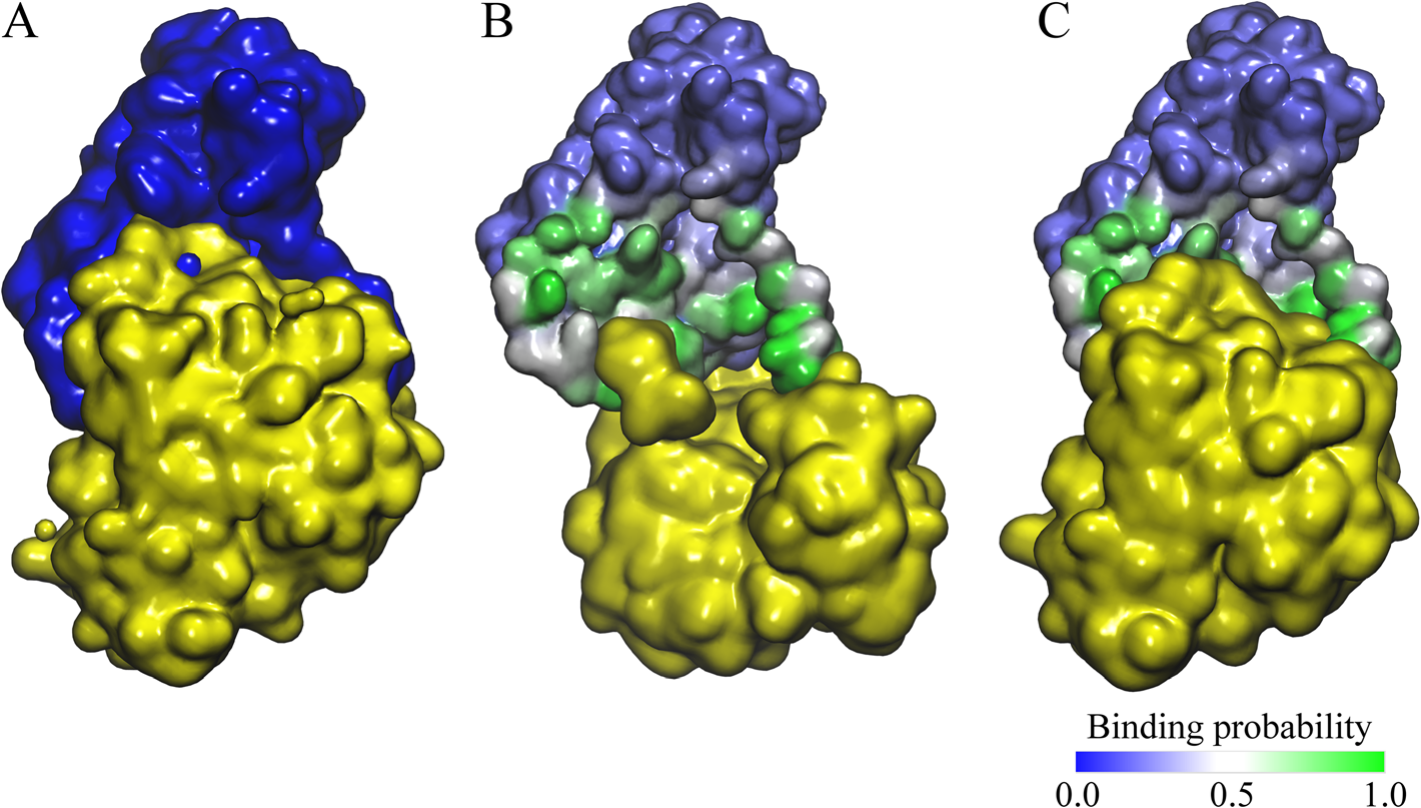
Model ranking for ARAT homo-dimer. The experimental complex structure is shown in (**a**) with the chain A colored in blue and the chain B colored in yellow. The top ranked models by ZDOCK and *e*Rank^PPI^ are shown in (**b**) and (**c**), respectively. In **b**, **c**, the surface of the chain A is colored according to interface probability estimated by *e*FindSite^PPI^ with the scale given in the bottom right corner (*blue*/*white*/*green* for the high/intermediate/low probability)

We conclude this study discussing several examples that illustrate the key features of *e*Rank^PPI^. Figure 7 shows how predicted PPI site information helps improve the ranking of near-native models. The experimental structure of aromatic amino acid aminotransferase homo-dimer (ARAT, PDB-ID: 1ay4, chains A and B) [84] is presented in Fig. 7a. Figure 7b and c show selected docked conformations with residues in the receptor protein are colored according to the predicted probability to be at the interface (green and blue correspond to the high and low interfacial probability, respectively). Only a partial overlap between the predicted and docked interface is apparent in Fig. 7b as a large chunk of the predicted interface area is exposed to the solvent. This conformation has an iRMSD of 23.64 Å and was ranked 1^st^ by ZDOCK, whereas *e*Rank^PPI^ placed it at the rank 413. In contrast, the docked interface shown in Fig. 7c has a substantial overlap with that predicted by *e*FindSite^PPI^; the iRMSD of this model is 6.11 Å and it is ranked 1^st^ and 14^th^ by *e*Rank^PPI^ and ZDOCK, respectively.

**Fig. 8:**
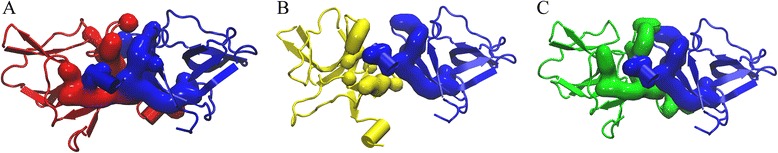
Model ranking for repressor protein cI homo-dimer. The experimental complex structure is shown in (**a**) with chain A colored in blue and chain B colored in red. The top ranked models by ZDOCK (*chain B is yellow*) and *e*Rank^PPI^ (*chain B is green*) are shown in (**b**) and (**c**), respectively. A cartoon representation is used for both chains with interface residues presented as a solid surface

Next, we present a case study that illustrates how contact-based symmetry improves the ranking of near-native models for homo-dimers. Figure 8a shows the crystal structure of λ repressor C-terminal domain (repressor protein cI, PDB-ID: 1f39, chains A and B) [85], whereas Fig. 8b and c present the top ranked conformations by *e*Rank^PPI^ and ZDOCK, respectively. The symmetry score implemented in *e*Rank^PPI^ ranges from 0 (no symmetry) to 1 (perfect symmetry); the native complex has a perfect symmetry as indicated by a CBS of 1.00. The top ranked model by ZDOCK has an iRMSD of 14.89 Å and a symmetry score of 0.00. The lack of symmetry is evident in Fig. 8b; *e*Rank^PPI^ placed this model at rank 806 because of the low CBS score. On the other hand, the top ranked model by *e*Rank^PPI^ shown in Fig. 8c has a high symmetry score of 0.85 and it is indeed the best model constructed for this target with an iRMSD of 1.27 Å. ZDOCK placed this model at rank 286, therefore, the symmetry score was critical to improve the ranking of this near-native conformation. We note that the contact-based symmetry score is not only intuitive as it ranges from 0 to 1, but also it can be calculated for any protein complex, including those constructed using computer-generated monomer structures.

**Fig. 9:**
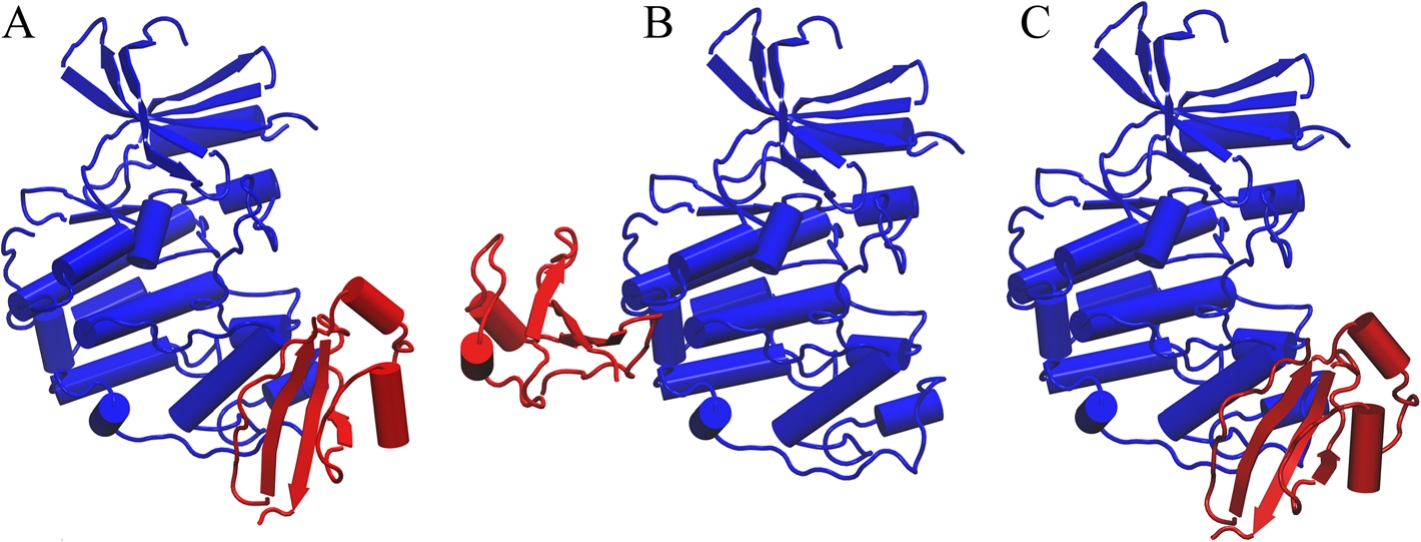
Model ranking for CDK2/CksHs1 hetero-dimer. The receptor (CDK2) and ligand (CksHs1) are colored in blue and red, respectively. **a** The experimental complex structure, **b** the top ranked model by ZDOCK, and **c** the nearest-native docked conformation

Finally, we discuss an example of the hetero-dimer complex between the human cyclin-dependent kinase 2 and cell cycle-regulatory protein CksHs1; the crystal complex structure is shown in Fig. 9a (CDK2, PDB-ID: 1buh, chains A and B) [86]. Figure 9b shows the structure of the top ranked conformation by ZDOCK, which has an iRMSD of 18.53 Å and was ranked 6^th^ by *e*Rank^PPI^. Figure 9c presents the structure of the nearest-native complex found within the set of 2000 conformations generated by ZDOCK that has an iRMSD of 0.98 Å. This model is ranked 28^th^ by ZDOCK, whereas *e*Rank^PPI^ placed it at rank 2. MCC of PPI site prediction for this target is only 0.39, nonetheless, despite the moderate accuracy of interface residue prediction, *e*Rank^PPI^ ranked this nearest-native conformation much higher than ZDOCK.

## Conclusion

In this study, we developed *e*Rank^PPI^, an algorithm for the selection of correct docking conformations constructed by rigid-body protein docking. *e*Rank^PPI^ features a new scoring function that integrates the predicted interface location with protein docking potentials and a contact-based symmetry score. Comprehensive benchmarking calculations show that *e*Rank^PPI^ has a high tolerance to structural imperfections in computer-generated protein models, therefore, it opens up a possibility to conduct the exhaustive structure-based reconstruction of PPI networks across proteomes.

## Availability of supporting data

The methods and datasets used in this study are available at www.brylinski.org/erankppi.

## Abbreviations

CBS: Contact-based symmetry score
LR: Linear regression
MCC: Matthew’s correlation coefficient
PCS: MCC-based pairwise contact score
PDB: Protein data bank
PPIs: Protein-protein interactions
SVMs: Support vector machines
